# The association between extreme heat and sperm quality in a chronically heat-exposed population: An observational study in Singapore

**DOI:** 10.1097/EE9.0000000000000533

**Published:** 2026-10-15

**Authors:** Samuel H. Gunther, Bing Yang Tan, Jason K. W. Lee, Shaili P. Sashidharan, Shiao-yng Chan, Qiong Wang, Zhongwei Huang

**Affiliations:** a Human Potential Translational Research Program, Yong Loo Lin School of Medicine, National University of Singapore, Singapore; b Campus for Research Excellence and Technological Enterprise (CREATE), 1 CREATE Way, Singapore; c Heat Resilience & Performance Centre, Yong Loo Lin School of Medicine, National University of Singapore, Singapore; d Department of Physiology, Yong Loo Lin School of Medicine, National University of Singapore, Singapore; e Department of Obstetrics and Gynaecology, National University Hospital, Singapore; f NUS Bia-Echo Asia Centre for Reproductive Longevity and Equality (ACRLE), Yong Loo Lin School of Medicine, National University of Singapore, Singapore; g Department of Obstetrics and Gynaecology, Yong Loo Lin School of Medicine, National University of Singapore, Singapore; h Institute for Human Development and Potential (IHDP), Agency for Science, Technology and Research (A*STAR), Singapore; i School of Public Health, Sun Yat-sen University, Guangzhou, China

**Keywords:** Extreme heat, Male fertility, Sperm quality, Spermatogenesis

## Abstract

**Background:**

Extreme heat has detrimental effects on male fertility. Previous studies conducted mainly in temperate countries have reported an association between higher ambient temperatures and decreased sperm quality. These findings might not be applicable to populations in tropical regions, who are chronically exposed to high ambient heat.

**Objectives:**

To address this knowledge gap, we measured the association between extreme heat during the spermatogenic life cycle and sperm quality in a population of 914 males who are 21–43 years old residing in a tropical country, Singapore.

**Methods:**

Medical records from 2011 to 2022 were analyzed alongside daily weather station records. Linear regression was used to estimate the association between the occurrence of days with maximum temperature >34.7°C during the 90-day spermatogenic life cycle and indicators of sperm quality. Sensitivity analyses were conducted for participants exposed to extreme heat multiple times during spermatogenesis.

**Results:**

Adjusting for age, race, body mass index, and days of abstinence before sample collection, exposure to extreme heat during the late stage of spermatogenesis (0–33 days before collection) was associated with lower sperm concentration (β = −9.3 million/mL, 95% confidence interval: –17, –1.8, *P* = 0.015). This amounted to a 20% reduction from the mean sperm concentration in the sample. Multiple (≥2) days of exposure to extreme heat in the late stage of spermatogenesis were associated with a larger reduction in sperm concentration (β = −12 million/mL, 95% confidence interval: −22, −2, *P* = 0.019).

**Conclusion:**

Even in tropical regions, with high ambient temperatures year-round, the occurrence of extremely hot days during the late stages of spermatogenesis is associated with lower sperm concentration. The degree of risk increases with more frequent exposures to extreme heat.

**What this study adds**

While previous research has linked ambient heat to decreased sperm quality, our study reveals that extreme heat adversely impacts male fertility even in populations chronically adapted to high temperatures, such as in Singapore. The occurrence of extremely hot days during the late stages of spermatogenesis is associated with lower sperm concentration, and multiple (≥2) days of exposure to extreme heat in the late stage of spermatogenesis were associated with a larger reduction in sperm concentration.

## Introduction

Climate change inflicts catastrophic damage on agriculture, industry, and economies worldwide. It also poses a threat to human health as global warming exposes more people to extreme heat. As detailed in the 2023 report of the *Lancet* Countdown on health and climate change, heat-related mortality in vulnerable populations increased by 85% in the years 2013 to 2022, compared with 1991 to 2000, while concurrent increases in climate change-induced floods and transmission of vector-borne diseases have also put the general population at increased risk of heat-related morbidity and mortality.^1^

The warming climate presents an acute threat to sexual and reproductive health, with sperm quality having been shown to suffer after exposure to extreme temperatures.^2–4^ Previous observational studies have reported that each 1°C increase in testicu.lar temperature is associated with a 14% decrease in the amount of spermatogenesis,^2^ while comparisons of men exposed and not exposed to heat have found a 7% reduction in sperm concentration, a 52% reduction in sperm count, a 93% reduction in sperm motility, and a 65% reduction in sperm progressive motility in the exposed group.^3^ In particular, occupational exposure that raises the temperature of the testes has been linked to reductions in sperm count and concentration in populations of industrial workers.^4^ Finally, the mental health toll of male infertility is emerging as an area of concern, with over 60% of participants in a recent study reporting that a diagnosis of infertility had impacted their mental health and caused them to experience more feelings of sadness and anxiety.^5^

There remains an important research gap, however, as the studies cited above were conducted in temperate regions with clear seasonal variations in weather, rather than in tropical regions where ambient temperatures and heat exposure are high year-round. Residents of these regions may have undergone heat acclimatization,^6^ shielding them to some degree from the impacts of extreme heat exposure by either conferring a greater degree of physiological heat resilience or ingraining protective behaviors such as constant use of personal cooling methods. Sperm health in the wider context of global declines in fertility has not been rigorously investigated, with reports instead focusing on demographic changes or positive developments such as wider access to contraception and increasing levels of women’s attainment.^7^ While these may be the main factors driving declining fertility in countries in higher stages of development, it is possible that the increasing frequency of extreme heat events will cause fertility rates to decline even further due to male subfertility, a hypothesis that is best modeled using clinical data from populations chronically exposed to heat.

Hence, this study sought to establish links between climate extremes in a tropical setting, namely Singapore, and the risk of low sperm quality, that is, low sperm concentration, count, motility, progressive motility, and vitality.

## Methods

### Study population

This study was conducted in Singapore, a tropical island city-state located one degree north of the equator, which experiences limited variation in ambient heat (average monthly temperatures range from 26.6°C to 28.2°C; relative humidity ranges from 64% to 95%).^8^ Since the nation’s independence in 1965, Singapore has been experiencing a steady decrease in total fertility rate, recording a historic low of 0.87 live births per female in 2025,^9^ thus making low fertility and diminishing birth cohorts national concerns. The potential study population consisted of all husbands of couples, at least 21 years of age, who submitted a semen sample to ascertain a diagnosis of male infertility to the National University Hospital, Singapore, andrology laboratory from July 2011 to February 2022 (n = 914). Those undergoing fertility treatment, chemotherapy, or other gonadotoxic treatments were excluded to avoid excessive bias due to nonenvironmental factors.

### Semen sample collection

Participants were advised on a standard method of semen collection by masturbation and were further instructed to abstain from ejaculation from 3 to 6 days before submitting their sample into a sterile collection bottle provided by the andrology laboratory. Each participant submitted just one sample at one point. Samples were subjected to standard semen analyses based on the World Health Organization 2010 manual for performing semen analysis.^10^ Sperm concentration (million/mL), total number of sperm (million), total sperm motility (%), total sperm progressive motility (%), and sperm vitality (%) were the key parameters measured. Several other demographic factors were recorded at the time of sample collection, namely age (years), race (Chinese, Malay, Asian Indian, or Other, i.e., not belonging to the other three groups; in Singapore, this information is listed on residents’ identification cards and routinely recorded during biomedical research studies), body mass index (BMI; kg/m^2^), and residential address (street name and number), as well as number of days of abstinence before semen analysis.

### Extreme heat exposures

Daily meteorological records stored and maintained by the Singapore National Environment Agency (NEA)’s Meteorological Service Singapore (Ministry of Sustainability and the Environment, Republic of Singapore) were used to determine the occurrence of extreme heat.^11^ Daily climate conditions from 1980 to the present are recorded at 20 weather stations spread evenly across the main island of Singapore and its minor outlying islands. Figure 1 displays the approximate locations of the NEA weather stations and their proximity to the residential addresses of the participants.

**Figure 1.**
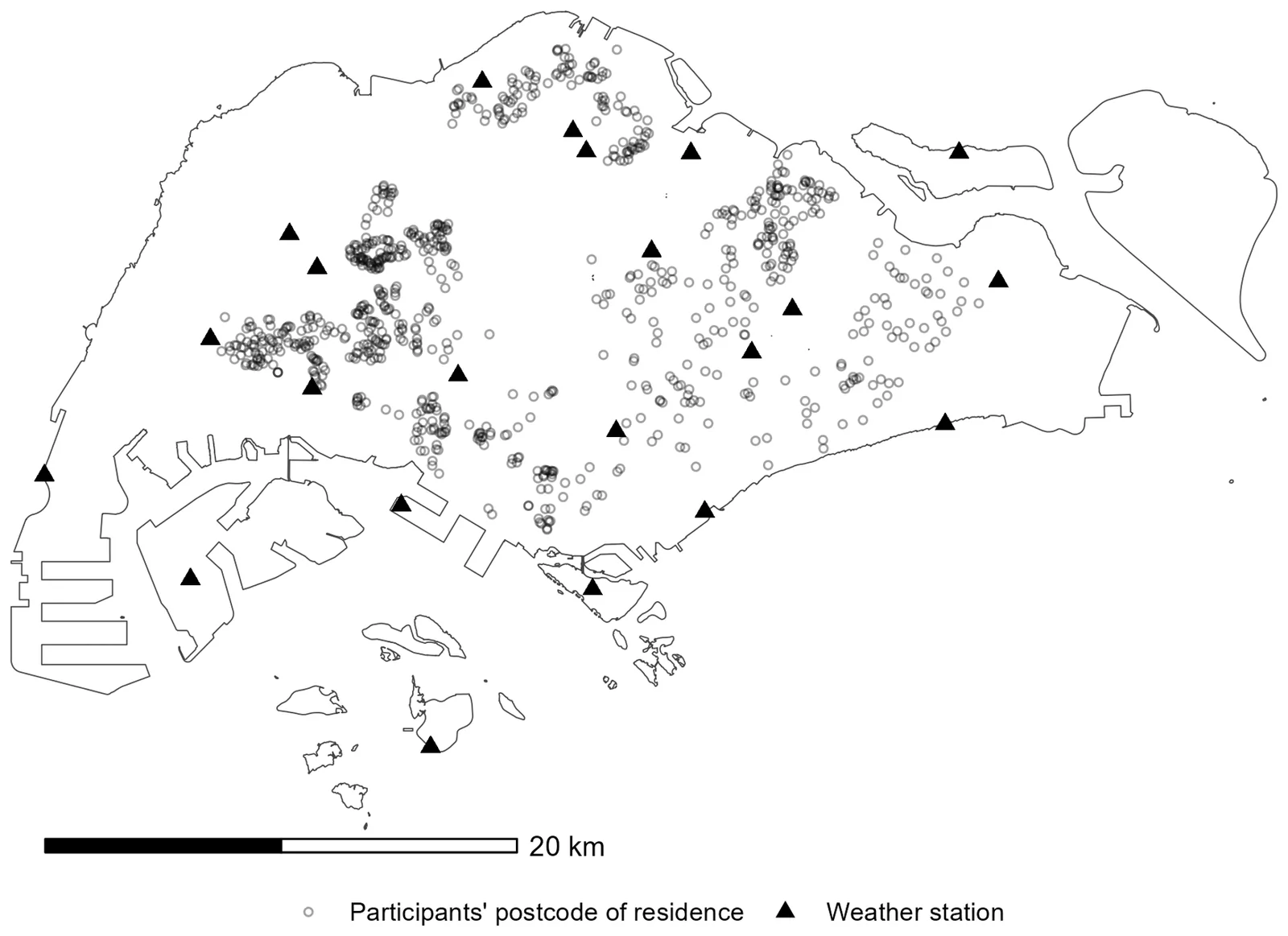
Map of participants’ residential addresses relative to weather stations currently recording daily temperature parameters in Singapore.

Overall exposure during the entire 90-day spermatogenic life cycle was analyzed, and we studied two exposure periods—0–33 days and 34–77 days before sample collection—as representing late-stage and early-stage sperm development, respectively.^10^

For this study, extreme heat was defined as the occurrence of any one day with a maximum temperature exceeding the 99th percentile of historical maximum temperatures in Singapore based on ERA5 climate reanalysis data, determined to be 34.7°C. The 99th percentile cutoff is a statistical definition that has been used in previous studies of environmental impacts on sperm quality,^10,11^ though it should be noted that the 95th percentile cutoff is sometimes used in temperate settings.^12^ To measure exposure to extreme heat, participants’ residential addresses were linked to temperatures that were interpolated from weather station observations using inverse distance weighting. While laboratory staff did not verify whether participants actually stayed at their reported address, in Singapore it is unlawful to report a false address or fail to report a change in address in a timely manner; hence, it is likely that recorded addresses were accurate. Daily maximum temperatures (in °C) from each respective weather station for the 90 days preceding the participant’s semen sample collection date were accessed. The key exposure windows defined above were then assessed to determine whether the maximum temperature exceeded 34.7°C on any day within them; if so, the participant was considered to have been exposed during that window.

### Statistical analyses

Linear regression was used to model the association between exposure to extreme heat during each exposure window and five key indicators of sperm quality as defined by the World Health Organization 2010 guidelines—concentration, count, motility, progressive motility, and vitality. Models were adjusted for participant age, race, BMI, and number of days of abstinence before semen analysis. The adjustment for race was based on the likely differential levels of occupational heat exposure between racial groups, as Malay and Indian males in Singapore are more likely to work in outdoor or physical labor-intensive fields, such as security and transportation, compared with Chinese males.

One linear regression included an indicator variable for any exposure to extreme heat within the 90-day period before sample collection, and a separate linear regression included two indicator variables—one for exposure in the 0–33 day (late) period before collection, and one for exposure in the 34–77 day (early) period. We adjusted each indicator variable’s *P* value using the Holm-Bonferroni method to account for the multiple comparisons in the analysis, and for the regressions with two indicator variables, we also conducted a joint F test of the significance of the two variables. We also conducted a sensitivity analysis where the indicator variables measured exposure to multiple (at least 2) extreme heat days in the same periods. The regression analysis described above was performed for these groups.

All quantitative analyses were performed using R version 4.5.1 (R Foundation for Statistical Computing, Vienna, Austria). The cutoff for statistical significance was set at 0.05, and that for marginal significance was set at 0.10.^13^

### Ethical approval

Ethical approval to access the anonymized andrology laboratory records was obtained from the National Healthcare Group Domain Specific Review Board (Ref: 2021/00131). As the medical records were completely anonymous, informed consent from the participants was not required.

## Results

### Participant demographics and outcomes

Sociodemographic characteristics and semen parameter outcomes of the 914 study participants are summarized in Table 1. Consistent with deriving these participants from specialized fertility clinic patient populations, the proportion with low sperm quality—especially total motility and vitality—was higher than would be expected in a healthy general population.

**Table 1.** Sociodemographic characteristics and semen parameter outcomes of the study population (n = 914).

Age, mean (SD)	32.2 years (2.2)
Race, n (%)	
Chinese	557 (0.6)
Malay	158 (0.2)
Indian	115 (0.1)
Other races	84 (0.1)
Body mass index, mean (SD)	26.3 kg/m^2^ (5)
Days of abstinence before semen analysis, median (range)	5 (0–56)
Sperm concentration, mean (SD)	47.5 million/mL (40.8)
Categorized by WHO 2010 guidelines, n (%)	
Normal (≥15)	679 (74.5)
Low (<15)	233 (25.5)
Sperm count, mean (SD)	156.3 million (175.9)
Categorized by WHO 2010 guidelines, n(%)	
Normal (≥39)	670 (75)
Low (<39)	223 (25)
Sperm total motility, mean (SD)	40% (17.2)
Categorized by WHO 2010 guidelines, n (%)	
Normal (≥40)	461 (53.8)
Low (<40)	396 (46.2)
Sperm progressive motility, mean (SD)	38% (30.5)
Categorized by WHO 2010 guidelines, n (%)	
Normal (≥32)	544 (67.7)
Low (<32)	260 (32.3)
Sperm vitality, mean (SD)	55.8% (15.6)
Categorized by WHO 2010 guidelines, n (%)	
Normal (≥58)	390 (49.8)
Low (<58)	393 (50.2)

Counts do not always add up to 914 due to missing values.

SD indicates standard deviation; WHO, World Health Organization.

### Association between extreme heat and sperm quality

The association between the occurrence of extremely hot days (maximum temperature >34.7°C) and sperm quality parameters for the overall 90-day period and the 0–33 and 34–77 days before sample collection are depicted in Panels A and B of Table 2, respectively. Results are shown for all participants and reflect the multivariable models only, adjusted for age, race, BMI, and days of abstinence before sample collection. The raw means of the sperm quality parameters and the differences between exposed and nonexposed participants are tabulated in Supplementary Table S1; https://links.lww.com/EE/A486.

**Table 2.** Associations between exposure to ≥1 day of maximum temperature >34.7°C in different exposure windows and sperm quality parameters in the overall study population, adjusted for age, race, body mass index, and days of abstinence before sample collection.

Exposure window	Concentration (million/mL)		Count (million)	Motility (%)	Prog. Motility (%)	Vitality (%)
Panel A: Exposure window is 0–90 days before sample collection						
0–90 days	β	−3.3	−5.1	0.96	−0.36	2.5
	95% CI	(−8.9, 2.4)	(−26, 16)	(−1.8, 3.8)	(−3.4, 2.6)	(−0.074, 5.2)
	*P*	0.26	0.63	0.5	0.81	0.057
	Adj. *P*	1	1	1	1	0.29
	n	909	890	854	801	780
Panel B: Exposure windows are 0–33 and 34–77 days before sample collection						
0–33 days	β	−9.3	−18	−0.75	−0.31	2.3
	95% CI	(−17, −1.8)	(−46, 9.3)	(−4.7, 3.3)	(−4.1, 3.4)	(−1.5, 6.1)
	*P*	0.015	0.2	0.71	0.87	0.23
	Adj. *P*	0.076	0.78	1	1	0.78
34–77 days	β	5.6	19	4	1.5	2.7
	95% CI	(−2.1, 13)	(−7.5, 46)	(0.43, 7.6)	(−2, 5)	(−0.79, 6.2)
	*P*	0.15	0.16	0.028	0.39	0.13
	Adj. *P*	0.51	0.51	0.14	0.51	0.51
	F test *P*	0.039	0.24	0.081	0.69	0.045
	n	909	890	854	801	780

Results are depicted as linear regression coefficient (β), followed by 95% confidence interval, followed by *P* value calculated using heteroskedasticity-robust standard errors, and finally by *P* value adjusted for multiple comparisons using the Holm-Bonferroni procedure. Differing numbers of observations reflect different frequencies of missing values in the dependent variables.

CI indicates confidence interval.

We observed that the occurrence of at least one extremely hot day during the 0–33 day period before sample collection was significantly associated with lower sperm concentration (β = −9.3 million/mL, 95% confidence interval: −17, −1.8, *P* = 0.015). The estimated coefficient of −9.3 million amounted to about 20% of the mean sperm concentration in the sample. This association remained marginally significant (*P* = 0.076) after Holm-Bonferroni adjustment for multiple comparisons. This was due to the multiple comparison adjustment reducing statistical power,^14^ combined with high unexplained variance in the dependent variable, as only a limited number of demographic variables were collected from participants and included as regression covariates. No other parameter was significant at any conventional level after adjustment.

We observed that a subset of participants was exposed to extreme heat at multiple points during the 90 days before sample collection, mostly during 2 or 3 days of consecutive high temperatures. These included 66 participants exposed multiple times during the late-exposure period (0–33 days) and 52 during the early-exposure period (34–77 days). The association of multiple heat exposures with sperm quality parameters is tabulated in Table 3, adjusted for age, race, BMI, and days of abstinence before sample collection. The raw means of the sperm quality parameters and the differences between exposed and nonexposed participants are tabulated in Supplementary Table S2; https://links.lww.com/EE/A486.

**Table 3.** Associations between exposure to multiple (≥2) days of maximum temperature >34.7°C in different exposure windows and sperm quality parameters in the overall study population, adjusted for age, race, body mass index, and days of abstinence before sample collection.

Exposure window	Concentration (million/mL)		Count (million)	Motility (%)	Prog. Motility (%)	Vitality (%)
Panel A: Exposure window is 0–90 days before sample collection						
0–90 days	β	−6.3	−7.1	0.37	−1.1	1.5
	95% CI	(−13, 0.4)	(−32, 18)	(−3.3, 4)	(−4.6, 2.5)	(−2, 4.9)
	*P*	0.066	0.58	0.84	0.56	0.41
	Adj. *P*	0.33	1	1	1	1
	n	909	890	854	801	780
Panel B: Exposure windows are 0–33 and 34–77 days before sample collection						
0–33 days	β	−12	−12	−3.9	−4.3	−1.1
	95% CI	(−22, −2)	(−52, 28)	(−10, 2.4)	(−10, 1.5)	(−7.6, 5.4)
	*P*	0.019	0.55	0.23	0.15	0.74
	Adj. *P*	0.094	1	0.69	0.6	1
34–77 days	β	1.3	3.2	5.5	3.4	4.2
	95% CI	(−7.4, 10)	(−29, 36)	(0.82, 10)	(−0.85, 7.7)	(−0.0084, 8.5)
	*P*	0.76	0.85	0.021	0.12	0.051
	Adj. *P*	1	1	0.11	0.35	0.2
	F test *P*	0.05	0.83	0.056	0.16	0.12
	n	909	890	854	801	780

Results are depicted as linear regression coefficient (β), followed by 95% confidence interval, followed by *P* value calculated using heteroskedasticity-robust standard errors, and finally by *P* value adjusted for multiple comparisons using the Holm-Bonferroni procedure. Differing numbers of observations reflect different frequencies of missing values in the dependent variables.

CI indicates confidence interval.

We observed that the association between multiple extreme heat exposures in the late period and low sperm concentration was stronger but estimated less precisely (β = −12 million/mL, 95% confidence interval: −22, −2, *P* = 0.019) compared with when both single and multiple exposures were considered. The reduced precision was due to the smaller number of participants experiencing multiple exposures. Again, this association remained marginally significant (*P* = 0.094) after adjustment for multiple comparisons, while no other parameter was significant at conventional levels after adjustment.

## Discussion

Our study represents one of the first investigations of the impact of extreme heat on sperm quality in a tropical climate defined by high ambient temperatures and little-to-no seasonal variation. It is therefore of interest that, despite chronic heat, we still observed differential effects of extremely hot days across different exposure periods. Specifically, we found that the occurrence of extreme heat within 0–33 days of sample collection was associated with significantly lower sperm concentration. We also found that being exposed to extreme heat multiple times during this period strengthened this association.

Our study approach, particularly in terms of the exposure periods that were analyzed, was most similar to a previous observational study conducted in China.^10^ Notably, there was some agreement in the key finding that temperature affected sperm concentration despite the different climatic conditions (temperate China and tropical Singapore). Lower concentrations, along with count and motility, were also found to be significantly associated with high temperatures in another study of 6 provinces in China.^15^ Unlike these studies, however, we further identified the late-stage development period (0–33 days before sample collection) as the exposure window in which these effects manifest. Regardless of the specific window, our findings are noteworthy as they suggest our study population may still be vulnerable to the physiological impacts of extreme heat despite their chronic exposure to high ambient temperatures, indicating that the effectiveness of heat acclimatization may be limited for certain physiological outcomes such as reproductive health.

Overall, our study agrees with the findings of the above-mentioned studies and several others which consistently reported an association between heat exposure and decreased sperm quality.^3,16–19^ Physiologically, this is believed to occur as the optimal temperature of the testicles is 2–4°C lower than core body temperature, with heat stress translating directly to increased testicular tissue temperature, which in turn results in decreased sperm concentration, motility, and viability.^3,16^

Abnormal semen quality has been implicated as a contributing factor in about half the cases of infertile or subfertile couples.^20^ These couples face challenges achieving natural pregnancy, contributing to marital stress and conflict, while men experiencing subfertility see it as a threat to their masculinity and avoid fertility consultations, thereby exacerbating the problem.^15^ It has also been reported that sperm quality is a marker of general male health and that men with poor semen quality—in particular, low sperm count—have a higher risk of metabolic syndrome, low testosterone, and hospitalization from cardiovascular disease and diabetes, independent of age.^21^ While previous studies have indicated that this association is mediated by hypogonadism,^21^ it is possible that extreme and chronic heat stress may also be involved, as we found extreme heat exposure to be associated with low sperm count. A related issue is the role of physical activity in promoting overall health, including sperm health.^22^ As the ambient environment warms to the point that it becomes unsafe to exercise outdoors due to an increased risk of heat stress, sperm quality could suffer from both the high heat and lower activity levels. Future studies linking sperm quality and general health should consider the potential role of heat stress in the analyses.

Caution is warranted in generalizing our findings to a wider population for several reasons. It is possible that study participants, aware of their upcoming clinic appointment, modified their behavior in the days immediately preceding the appointment in such a way as to protect their testes from unnecessary heat exposure, especially during heatwave periods when extreme heat persisted over multiple consecutive days. Protective behaviors have been previously described and include wearing looser-fitting underwear, sitting for shorter periods, bathing and sleeping in cooler temperatures, as well as more innate behaviors such as avoiding spending time outdoors in extreme heat. Each of these behaviors has been shown to be associated with improvements in sperm quality parameters. Similarly, participants who experienced an extreme heat event long before their clinic appointment may have adopted protective behaviors so that when they experienced another extreme heat event closer to their appointment, they were able to reduce the physiological impact. As the only behavioral “control” was to request that participants practice abstinence for at least 3–6 days before their appointment, the impact of other behavior changes cannot be excluded as a potential explanation.

A further limitation of our study was that there was no way of precisely measuring the degree of exposure to the extreme heat events that occurred. This is a common limitation in observational studies of environmental exposures, and we attempted to address it by linking temperature data with participants’ residential location. It remains possible that participants were either exposed to extreme heat in outdoor locations at their workplace or elsewhere some distance away from their home. That said, Singapore’s temperatures tend to be spatially homogeneous due to the country’s small size and consistent elevation, minimizing the chance that temperatures experienced outside the home would be drastically different. Participants may also have spent more time indoors or increased their use of personal cooling methods such as air conditioning on hot days, thereby minimizing their actual exposure to the heat and attenuating potential associations. Future studies may consider innovative methods of directly measuring environmental exposures outside the laboratory setting.

Another direction of improvement for future studies would be to sample the sperm quality parameters from the same individuals at multiple time points. This additional data would improve the analysis by allowing the researcher to observe intraindividual variability and separate this from the effect of extreme heat exposure. The statistical power of the analysis could also be improved by increasing the number of participants and collecting more demographic and lifestyle information such as occupation, smoking, and alcohol use for inclusion in the regression models.

It should also be emphasized that our participants were derived from a population of fertility clinic patients, some of whom were seeking treatment for fertility problems. Hence, their sperm quality (particularly sperm vitality) may have already been impaired for reasons unrelated to heat exposure. We mainly observed impacts of extreme heat on sperm vitality, which could indicate that heat impacts manifest strongest in severely compromised sperm, and that our results may not be representative of the general reproductive-age population. While we did not observe longitudinal declines in sperm quality in our study sample, it is possible that our results were impacted by a general decline in sperm quality over the study period. Finally, there remained the potential for our findings to be affected by unmeasured confounders. Cigarette smoking, alcohol intake, dietary practices, physical activity, psychological stress, lack of sleep, and exposure to electromagnetic radiation from mobile phones have all been cited as potentially impacting sperm health,^23–25^ but these were not recorded in the andrology laboratory records. Furthermore, while exposure to air pollution has also been implicated in declining sperm quality,^26^ air pollution data were not recorded by NEA weather stations at the time of the study.

Our results suggest that men who are planning to conceive soon (1–3 months) should be discouraged from unnecessarily going outdoors on extremely hot days. Those who have no option but to expose themselves to extreme heat should also be encouraged to cool their testes, either by wearing loose pants and underwear or sleeping in cooler temperatures, in the days immediately preceding their and their partner’s attempt at conceiving. For these men, institutional changes such as allowing more flexible work-from-home options or assigning tasks that minimize the amount of time spent outdoors could also be beneficial. As the climate continues to warm, these and other interventions should be encouraged to achieve tangible, positive outcomes in reproductive health in the tropics and beyond.

## Conflicts of interest statement

The authors declare that they have no conflicts of interest with regard to the content of this report.

## ACKNOWLEDGMENTS

The authors would also like to thank Ms. Gabriel May Moe Kyaw for her contributions to the figures used in this article.

Daily ambient temperature data for Singapore weather stations are accessible at the Singapore National Environment Agency’s website: (https://www.weather.gov.sg/home/). Anyone who wishes to access the data may freely download the relevant monthly temperature files in either.csv or .pdf format and concatenate them into annual daily temperature datasets. ERA5 climate reanalysis data are accessible at https://cds.climate.copernicus.eu/datasets/reanalysis-era5-single-levels?tab=overview.

Individual participant demographic and outcome data that underlie the results reported in this article, after de-identification, as well as the study protocol, statistical analysis plan, and analytic code, will be made available beginning three months and ending five years following article publication to researchers who provide a methodologically sound proposal, for the purpose of achieving aims in the approved proposal. Proposals should be directed to the corresponding author: obgzwh@nus.edu.sg; to gain access, data requestors will need to sign a data access agreement.

## Supplementary Material
