# Supplementary material for: The association between extreme heat and sperm quality in a chronically heat-exposed population: An observational study in Singapore
Source: Environ Epidemiol. 2026 Oct 15;10(6):e533. doi: 10.1097/EE9.0000000000000533 (PMC13674966; doi:10.1097/EE9.0000000000000533)
Supplement: Supplementary file 1 [file ee9-10-e533-s001.pdf]

Supplementary Tables

Supplementary Table S1: Differences in means of sperm quality parameters between participants exposed and not exposed to ≥1 day of maximum temperature >34.7°C in different exposure windows.

		Concentration (million/mL)	Count (million)	Motility (%)	Prog. Motility (%)	Vitality (%)
0-90 days	Not exposed (n=707)	48.6	160	39.8	38.1	55.3
	Exposed (n=207)	43.8	145	40.5	37.4	57.6
	Difference	-4.77	-14.8	0.732	-0.739	2.25
	p	0.1	0.19	0.61	0.67	0.09
0-33 days	Not exposed (n=803)	48.5	159	39.9	38	55.5
	Exposed (n=111)	40.2	140	40.2	37.7	58.4
	Difference	-8.26	-19	0.249	-0.246	2.86
	p	0.022	0.17	0.9	0.9	0.12
34-77 days	Not exposed (n=803)	47.4	156	39.6	37.9	55.5
	Exposed (n=111)	48.3	158	42.8	38.5	58.4
	Difference	0.892	1.62	3.2	0.665	2.97
	p	0.81	0.9	0.067	0.73	0.074

Supplementary Table S2: Differences in means of sperm quality parameters between participants exposed and not exposed to multiple (≥2) of maximum temperature >34.7°C in different exposure windows.

		Concentration (million/mL)	Count (million)	Motility (%)	Prog. Motility (%)	Vitality (%)
0-90 days	Not exposed (n=792)	48.4	158	40	38.2	55.7
	Exposed (n=122)	41.7	145	39.8	36.5	56.8
	Difference	-6.71	-13.4	-0.137	-1.72	1.15
	p	0.046	0.31	0.94	0.38	0.51
0-33 days	Not exposed (n=862)	48.1	157	40.1	38.2	55.8
	Exposed (n=52)	36.7	144	37.7	34.5	56
	Difference	-11.4	-13.4	-2.41	-3.65	0.214
	p	0.024	0.49	0.43	0.21	0.95
34-77 days	Not exposed (n=848)	47.7	157	39.7	37.9	55.6
	Exposed (n=66)	45.2	148	43.4	39.1	59.1
	Difference	-2.42	-9.21	3.67	1.23	3.56
	p	0.54	0.56	0.1	0.58	0.064
